# Closed-leg standing long leg radiographs can be a useful tool to assess whether the joint line is parallel to the ground in restricted kinematic alignment total knee arthroplasty

**DOI:** 10.1186/s40634-023-00606-y

**Published:** 2023-04-11

**Authors:** Takashi Kobayashi, Kazumi Goto, Masayoshi Otsu, Kazuhiko Michishita

**Affiliations:** 1 Department of Orthopedic Surgery, Yugawara Hospital, 2-21-6, Chuo, Yugawara, Kanagawa 259-0301 Japan; 2 Department of Orthopedic Surgery, Mitsui Memorial Hospital, 1, Kandaizumi-Cho, Chiyoda-Ku, Tokyo, 101-8643 Japan

**Keywords:** Knee arthroplasty, Total knee replacement, Kinematics

## Abstract

**Purpose:**

Restricted kinematic alignment (rKA) is a modified technique of kinematic alignment (KA) total knee arthroplasty (TKA) for patients with an outlier or atypical knee anatomy, striving to preserve the native knee joint line parallel to the ground in a bipedal stance. This study aimed to evaluate the accuracy of rKA TKA with a computed tomography (CT)-based patient-specific instrument (PSI) to achieve the preoperative plan with the joint line parallel to the ground level.

**Methods:**

Using a CT-based PSI, 74 closed-leg standing long-leg radiographs were obtained before and after rKA TKA. The hip-knee-ankle angle (HKA), joint line orientation angle (JLOA), lateral distal femoral angle (LDFA), and medial proximal tibial angle (MPTA) were measured. Bone resection accuracy was evaluated by postoperative HKA deviations from the planned alignment and joint line by postoperative JLOA deviations from the ground level.

**Results:**

The mean postoperative JLOA and HKA were 2.1° valgus (range, standard deviation: 6.0° valgus to 3.0° varus, 2.0) and 2.6° varus (3.5° valgus to 12.5° varus, 3.2), respectively. Postoperative JLOA and HKA were within ± 3° of the planned alignment for 69% and 86% of cases, respectively.

**Conclusions:**

Despite a static verification, we clarified how the joint line after rKA TKA was reproduced in the closed-leg long leg radiographs to mimic the limb position during gait. However, this imaging method is not well-established, and lack of long-term survivorship and the relationship between joint line inclination and clinical outcomes represented limitations of this study.

**Level of evidence:**

Level IV.

## Background

Kinematic alignment (KA) and restricted kinematic alignment (rKA) are alternative procedures for total knee arthroplasty (TKA), conventionally represented by the mechanical alignment (MA) technique that creates a neutral lower limb alignment by cutting the distal femoral and proximal tibia bones perpendicular to the mechanical axes. KA aims to generate a more physiological prosthetic knee by restoring the native knee anatomy and physiological soft-tissue balance [1–3]. KA involves aligning the femoral component on the cylindrical axis, performing anatomical rather than mechanical bone cuts, and no-soft-tissue release is required [4]. An early- to mid-term low complication rate and high function and satisfaction after KA TKA have been reported [5]. RKA is a modified technique of KA suggested by Vendittoli et al., as the range of alignment which would result in low wear and long-term implant success for the current TKA design remains unknown [6–8]. During rKA, an algorithm is used to adjust extreme patient anatomy that might be unsuitable for long-term implantation [6, 8].

KA TKA can be achieved using various techniques such as a magnetic resonance imaging-based patient-specific instrument (PSI), manual methods, and new technologies recently represented by computer-assisted surgery [7, 9–15]. In the late 2010s, computed tomography (CT)-based PSIs for KA were introduced, and have since been reported to improve the accuracy of bone cutting [16–19].

A recent study reported that joint line orientation in the coronal plane of the native knee was parallel to the ground and perpendicular to the weight-bearing axis of the limb in a bipedal stance, regardless of the presence of constitutional varus [20]. Because bipeds must place each foot directly beneath their center of body mass during single support, an angulation of the knee is required, i.e., the normal valgus position of the knee, in which the proximal tibia joint line is in slight varus to bring the tibial joint line more parallel to the ground [21]. Although bone-cutting accuracy and lower extremity alignment have been evaluated for each KA technique [9, 11–13, 22, 23], assessment of the joint line in the bipedal position has not been well studied. Few reports investigated joint line orientation after KA TKA using long-leg standing radiographs [14, 24]. Moreover, there have been no reports of joint line orientation using long-leg standing radiographs after rKA TKA with a CT-based PSI. Therefore, this study aimed to evaluate the accuracy of rKA TKA with a CT-based PSI to achieve the preoperative plan and a joint line parallel to the ground level. We hypothesized that the planned lower limb alignment and joint line would be obtained in rKA TKA using a CT-based PSI.

## Methods

### Study design

**Fig. 1:**
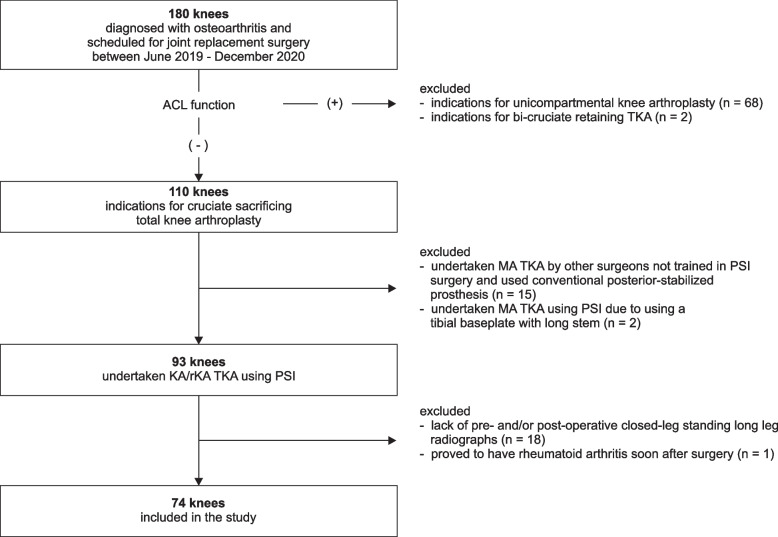
Flowchart of inclusion and exclusion criteria. The inclusion criteria for this study were patients who had undergone primary TKA using the rKA method with CT-based PSI and with pre- and post-operative long-leg radiograph taken at our facility. Patients with rheumatoid arthritis, who had undergone arthroplasty using conventional alignment methods or other technology assistance, and without long-leg radiographs were excluded. Abbreviations: ACL: anterior cruciate ligament; KA: kinematic alignment; MA: mechanical alignment; PSI: patient-specific instrument; rKA: restricted kinematically alignment; TKA: total knee arthroplasty

This study, which described a case series of accuracy validation of rKA TKA without a control group, was approved by our institutional review board. RKA TKA was introduced to our department by a single surgeon in June 2019. The radiographic results of 180 consecutive patients (41 men and 139 women), who were diagnosed with osteoarthritis of the knee from June 2019 to December 2020, were retrospectively reviewed. The inclusion criteria for this study were patients who had undergone primary TKA using the rKA method with CT-based PSI and those with pre- and post-operative long-leg radiographs taken at our facility. Patients with rheumatoid arthritis, who had undergone unicompartmental knee arthroplasty, who had undergone TKA using conventional alignment methods or other technology assistance, and without long-leg radiographs were excluded (Fig. 1). Seventy-four knees (11 men and 63 women) were included in this study. The mean age of the patients was 77.5 (range, standard deviation [SD]: 61–88, 5.9) years.

### Surgical techniques

To manufacture patient-specific cutting blocks, preoperative CT scans, including parts of the femoral head, knee, and ankle, were performed with the patient in supine position and the leg maximally extended and unloaded, according to a standardized protocol (MyKnee®, Medacta International S.A., Castel San Pietro, Switzerland). The images were uploaded to the company’s website. The anatomical landmarks used for planning included the hip center (the center sphere that best approximates the femoral head), distal femur center (the center of the intercondylar notch corresponding to the most distal point of trochlea), proximal tibial center (the midpoint between the medial and lateral eminences), and ankle center (the center point of a line connecting the medial and lateral malleoli). The tibial slope was determined on the medial tibial plateau referencing the sagittal mechanical axis. The tibial sagittal plane was defined by the mechanical axis and the axis perpendicular to the tangent to the posterior margins of the medial and lateral articular surfaces of the tibial plateau and passing from the tibial center. The engineers created three-dimensional (3D) bone models of the knee, and the plan for cutting blocks was validated by the surgeon.

To obtain rKA in the coronal plane, the planning protocol for all patients was as follows: bone resection of the distal femur and proximal tibia to restore a physiological joint line, femoral component rotation parallel to the posterior condylar axis, femoral component fitting to the anterior cortex without notching, tibial component rotation oriented parallel to Akagi’s line, and tibial posterior slope between 0° and 5° according to the patient’s anatomy [25]. However, this procedure was modified if the planned coronal resection angle was outside the rKA target zone of either a postoperative hip-knee-ankle angle (HKA, within ± 3°) and/or independent femoral or tibial coronal cuts (within ± 5°). These procedural modifications were previously described as the rKA protocol [6, 8, 24]. In cases where the plan as per the rKA protocol predicted that implant placement would be difficult without soft tissue dissection even after osteophyte resection, that is, where the preoperative valgus stress radiographs showed no medial joint space due to severe varus deformity, protocol violation was exceptionally allowed and the tibial varus cut angle plan was increased.

All surgery were performed by one surgeon using the same cemented implants, designed as cruciate-substitute medial-pivot prosthesis (GMK® Sphere, Medacta International S.A., Castel San Pietro, Switzerland). A midline incision and a medial parapatellar approach were used. The posterior cruciate ligament was resected in all cases. While no patients required additional medial soft tissue release, the iliotibial band was released from the tibia in patients with valgus deformity. Bone resections of the femur and tibia were performed using patient-specific cutting blocks via a measured resection technique. The patella was resurfaced if degenerative changes in the lateral facet were evident. All components were implanted with cement.

### Radiographic evaluation

**Fig. 2:**
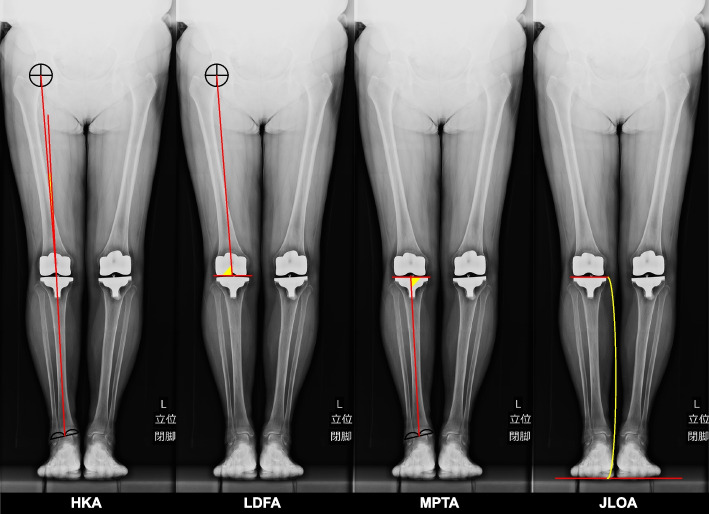
Measurements on closed-leg standing long leg radiographs. Abbreviations: HKA: hip-knee-ankle angle; JLOA: joint line orientation angle; LDFA: lateral distal femoral angle; MPTA: medial proximal tibial angle

All patients were evaluated preoperatively and postoperatively using closed-leg standing long leg radiographs. HKA, joint line orientation angle (JLOA), lateral distal femoral angle (LDFA) and medial proximal tibial angle (MPTA) were measured [24]. HKA describes the relationship between the mechanical axes of the femur and tibia as a deviation from 180° [1]. Negative values represent varus alignment, while positive values represent valgus alignment of the lower limb. JLOA describes the angle between the joint line and a line parallel to the floor, as reported previously [8]. LDFA was defined as the lateral angle between the femoral mechanical axis and the tangent line formed by the distal femoral condyles or components. MPTA was defined as the medial angle between the tibial mechanical axis and the tangent line formed by the tibial plateau or margin of the tibial component [26]. All measurements were performed by three readers (TK, MO, and KK) to assess inter-observer reproducibility as the manners described previously (examples are shown in Fig. 2).

The following evaluations were performed after the mean values of each measurement were obtained: (1) Coronal Plane Alignment of the Knee (CPAK) classification to describe preoperative knee phenotypes, where arithmetic HKA and joint line obliquity are calculated by the sum and difference of MPTA and LDFA as reported previously [27]; (2) scatter plots of preoperative LDFA and MPTA to determine the number of patients who needed a modified PSI plan for rKA TKA [25]; (3) the outliers from the rKA target zone of postoperative HKA (more than ± 3º), LDFA (more than ± 5º), and MTPA (more than ± 5º); (4) the outliers from the alignment target, which are defined as deviations of postoperative HKA, LDFA, and MPTA from the planned alignment, to analyze the accuracy of bone resections (outliers from alignment target were defined as deviations from the planned alignment of more than ± 3º [28]); and (5) deviations of the postoperative JLOA from the target of the ground level to investigate if a joint line parallel to the floor was achieved (outliers were defined as deviations from the floor more than ± 3º). The definition of outliers from the JLOA target has not been established because there have been no reports of JLOA measurement using closed-leg standing long-leg radiographs. In previous reports using standardized long-leg radiographs after KA TKA, postoperative JLOA was 1.0˚ ± 1.9˚ [24], and, even in a normal control, some cases had JLOA more than ± 2˚ in the ± 2SD range [14]. Thus, we defined the outliers from the JLOA target as deviations from the floor more than ± 3º in this study.

### Statistical analysis

A power analysis demonstrated that 41 patients would be required to detect a difference in measurements of 1.0º with a standard deviation (SD) of 2.0, a power of 80%, and an alpha error of 0.025. Mean and standard deviation angles for each measurement were computed. The percentage of the outliers from the rKA target zone and alignment target were also obtained. To assess the accuracy of PSI, the mean absolute error (MAE) between the planned and postoperative alignment was calculated. Inter-rater repeatability for each radiographic measurement were calculated using the inter-class correlation coefficient (ICC). All statistical analyses were performed with EZR (Saitama Medical Center, Jichi Medical University, Saitama, Japan), which is a modified version of R commander (version 4.2.1), a graphical user interface for R (The R Foundation for Statistical Computing, Vienna, Austria).

## Results

**Fig. 3:**
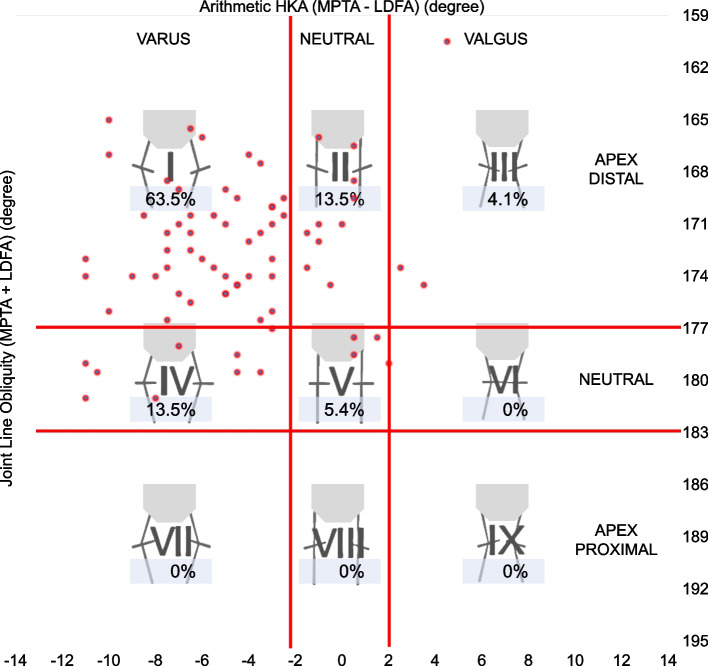
Plot of arithmetic hip-knee-ankle angle (HKA) against calculated joint line obliquity for the preoperative population included in this study, showing distribution by percentage of the nine Coronal Plane Alignment of the Knee (CPAK) phenotypes. The arithmetic HKA and joint line obliquity were calculated by the difference and sum of MPTA and LDFA as reported by MacDessi et al. [27]’s Coronal Plane Alignment of the Knee (CPAK) classification. Abbreviations: LDFA: lateral distal femoral angle; MPTA: medial proximal tibial angle

**Fig. 4:**
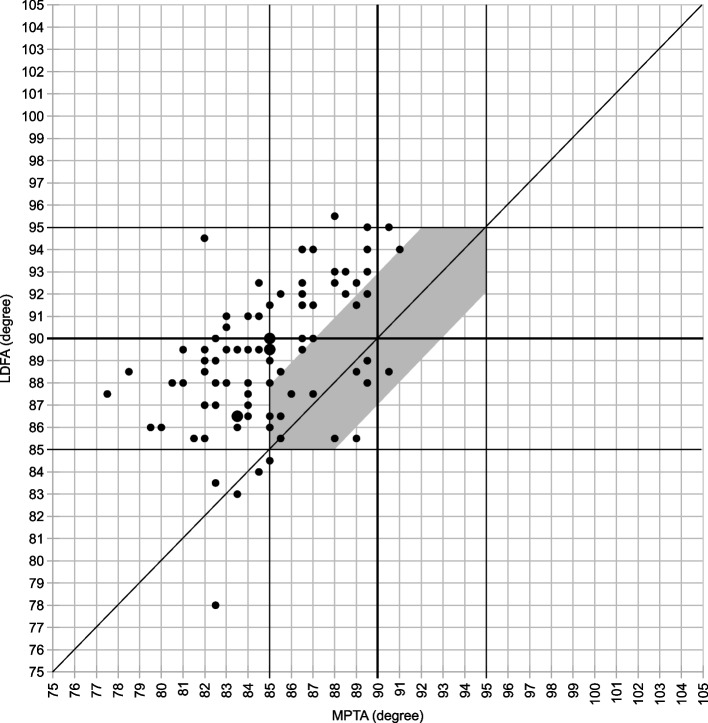
Scatterplots of preoperative LDFA and MPTA. The shaded areas represent patients in the rKA target zone during preoperative planning. Twenty percent of all cases are in this safety zone and the others required modifications of the cutting plans. Abbreviations: LDFA: lateral distal femoral angle; MPTA: medial proximal tibial angle

**Fig. 5:**
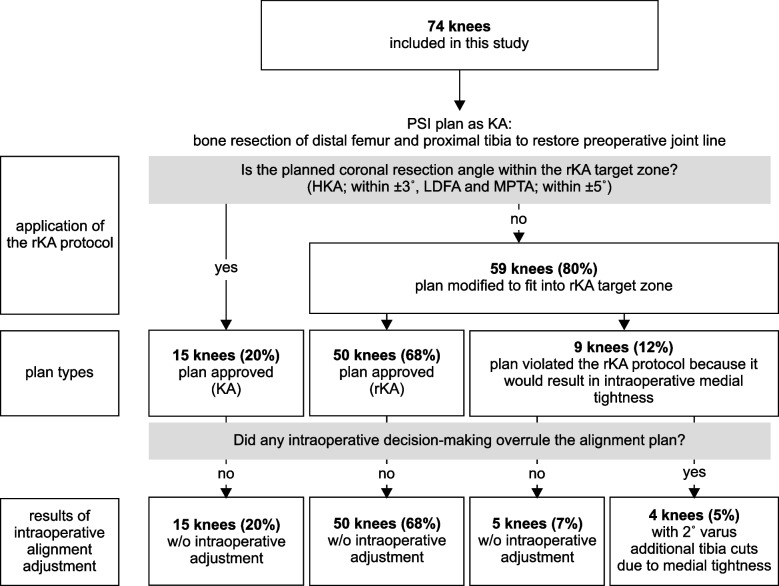
Flowchart showing the number of patients who had modifications to or deviated from the rKA plan and the results of intraoperative alignment adjustment Abbreviations: HKA: hip-knee-ankle angle; KA: kinematic alignment; LDFA: lateral distal femoral angle; MPTA: medial proximal tibial angle; PSI: patient-specific instrument; rKA: restricted kinematic alignment

**Fig. 6:**
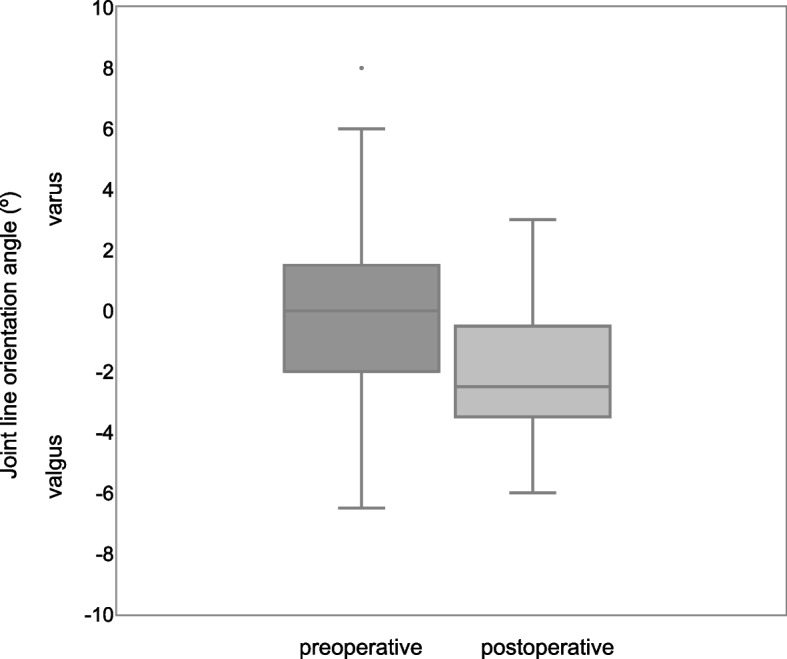
Preoperative and postoperative JLOA. Sixty-nine percent of the postoperative JLOA are within ± 3º of the floor. All outliers of the postoperative JLOA were in valgus orientation. Abbreviations: JLOA: joint line orientation angle

**Fig. 7:**
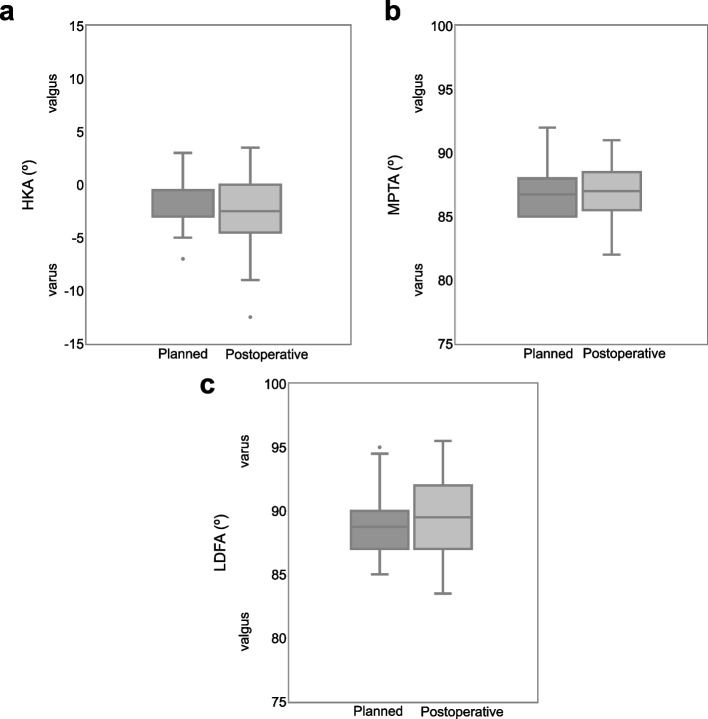
Distributions of postoperative HKA, MPTA, and LDFA compared to the planned alignment. **a** HKA. **b** MPTA. **c** LDFA. Abbreviations: HKA: hip-knee-ankle angle; LDFA: lateral distal femoral angle; MPTA: medial proximal tibial angle

The preoperative knee phenotypes of included patients by CPAK classification are shown in Fig. 3. The scatter plots of preoperative LDFA and MPTA are shown in Fig. 4. Figure 4 shows that 59 cases (80%) outside the rKA target zone required a modified PSI plan for rKA. There were 9 patients whose plan deviated from the rKA protocol, and 4 of these 9 patients needed 2˚ varus additional tibia cuts to the planned alignment; the decision flowchart is shown in Fig. 5. No patient required reversion to conventional instruments due to mismatches between PSI and anatomy. Preoperative, planned, and postoperative alignment measurements for each alignment category and the MAE between the planned and postoperative alignment are presented in Table 1. The mean postoperative JLOA was -2.1º (range, SD: 6.0º valgus to 3.0º varus, 2.0) and the mean postoperative HKA was -2.6º (3.5º valgus to 12.5º varus, 3.2). The MAE between the planned and postoperative HKA was 1.7º (range, SD: 0 to 7.7º, 1.6); LDFA, 1.6 (0 to 9º, 1.7); MPTA, 1.3 (0 to 5º, 1.0); and JLOA, 2.4 (0 to 6º, 1.6). The ICC between the planned and postoperative alignment were as follows: HKA: 0.61, 95%CI [0.45, 0.74]; LDFA: 0.65, 95%CI [0.50–0.77]; and MPTA: 0.61, 95%CI [0.44, 0.73]. The ICC of JLOA could not be calculated because the value of the plan to be compared was 0. The percentages of outliers from the rKA target zone and the alignment target are summarized in Table 2. Fifty-one patients (69%) showed postoperative JLOA within ± 3° of floor, and all outliers of postoperative JLOA were of valgus orientation (Fig. 6). Sixty-four (86%) of postoperative HKA was within ± 3º of the alignment target. In contrast, 2 patients (2.7%) showed a magnitude of error for postoperative HKA (deviations more than 3° from the alignment target). Thirty-four patients (46%) showed postoperative HKA deviations greater than ± 3°. The distributions of the postoperative HKA, LDFA, and MPTA compared to the planned alignment are shown in Fig. 7. The patella was resurfaced in 19 patients (26%).

**Table 1:** Measurements of preoperative, planned, and postoperative alignments and MAE between the planned and postoperative alignments

	Preoperative			Planned			Postoperative		
	Mean	Range	SD	Mean	Range	SD	Mean	Range	SD
HKA	-9.4	-24.0 to 6.5	5.7	-2.0	-7.0 to 3.0	1.9	-2.6	-12.5 to 3.5	3.2
					absolute error		1.7	0 to 7.7	1.6
LDFA	88.8	78.0 to 98.0	3.2	88.8	85.0 to 95.0	2.4	89.5	83.5 to 95.5	3.1
					absolute error		1.6	0 to 9.0	1.7
MPTA	84.1	77.5 to 90.5	2.6	86.8	85.0 to 92.0	1.7	86.9	82.0 to 91.0	2.1
					absolute error		1.3	0 to 5.0	1.0
JLOA	-0.1	-6.5 to 8.0	2.8	parallel to the floor			-2.1	-6.0 to 3.0	2.0
					absolute error		2.4	0 to 6.0	1.6

Varus measurements are negative, valgus measurements are positive in HKA. The tibial joint line slanted down to the medial side is expressed as a positive value in JLOA. *Abbreviations:* *HKA *Hip-knee-ankle angle, *JLOA *Joint line orientation angle, *LDFA *Lateral distal femoral angle, *MAE *Mean absolute error, *MPTA *Medial proximal tibial angle, *SD *Standard deviation

**Table 2:** Percentage of outliers from the rKA target zone and alignment target

	*Outliers from rKA target zone* (%)	*Outliers from alignment target* (%)
Measurements	Postoperative HKA more than ± 3º or LDFA/MTPA more than ± 5º	Postoperative measurement deviations from planned alignment more than ± 3º
HKA	46	14
LDFA	7	11
MPTA	14	5
JLOA	-	31

*Abbreviations:* *HKA* Hip-knee-ankle angle, *JLOA* Joint line orientation angle, *LDFA* Lateral distal femoral angle, *MPTA* Medial proximal tibial angle, *rKA* Restricted kinematic alignment

## Discussion

The most important finding of the present study was that the postoperative JLOA was within ± 3º of floor in closed-leg standing position in 69% of all patients undergoing rKA TKA using CT-based PSI. Although the concept of the KA method is to reproduce the physiological joint line of the normal knee [19], which is parallel to the floor during the swing phase of bipedal walking, the extent to which the joint line is tilted in the walking limb position has not been well-established. The current study provided a static verification; however, it notably also clarified how the reproduced joint line in the closed leg position mimicked the limb position during gait.

Two studies with techniques other than PSI previously investigated the postoperative joint line orientation using standardized long leg radiographs after KA TKA. The first study with optical computer navigation showed that the postoperative JLOA was 1.0º (2.6º valgus to 6.0º varus, 1.9) in 52 patients [24]. The second, with a cartilage probing technique, showed that the postoperative JLOA values were 0.6º ± 1.7º and 0.2º ± 1.1º in the KA and normal healthy control groups, respectively, whereas this value slanted down to valgus in the MA group [14]. Unlike these studies on KA, the mean postoperative JLOA and all outliers of postoperative JLOA in the current study had a valgus orientation. Potential factors contributing to the joint line tilting more valgus include the fact that the joint line was modified to be closer to the MA method in rKA compared to that in KA, and that on long-leg standing radiographs, the closed-leg position was closer to the adduction position of the lower leg during bipedal walking than was the open-leg position [21]. As the authors above reported, the joint line after KA TKA tended to varus on standardized long leg radiographs, and there is concern that this degree of varus may be excessive. However, a closed-leg position used in combination with the standardized long leg radiograph could be a simple optional tool to image the joint line during gait and assess that the joint line is not excessively varus inclined. However, there are no reports on the use of closed-leg standing long-leg radiographs. As such, this imaging method was not well-established and represented a limitation of this study.

Regarding the bone-cutting accuracy, 14% of patients in this study showed deviations from the planned HKA of more than ± 3º. The accuracy of CT-based PSI were only analyzed in a few studies [16, 18]. Pauzenberger et al. [18] demonstrated that the percentage of patients with HKA deviations greater than ± 3º was 10.1% and 25.8% in the CT-based PSI and the conventional groups, respectively. In a study of 25 patients with TKA using CT-based PSI, Ensini et al. [16] found that the outliers of component alignment were 17% and 9% for the femur and tibia, respectively. These findings were consistent with our present results. However, in two patients the error for postoperative HKA was more than 3° from the alignment target. This was due to violation of the plan per rKA protocol and an error of bone cutting and intraoperative decision making, specifically additional cutting of the tibia 2˚ varus with residual medial tightness. In addition, the 9 patients who violated the rKA protocol plan due to preoperative severe varus deformity showed significant error for postoperative HKA (range, SD: -12.5º to -4.0º, 2.7). Moreover, their MAE between planned and postoperative HKA was 2.4˚, which was higher than the overall MAE of 1.7˚. This fact could not be explained by the accuracy of the PSI alone because all postoperative HKA errors in these patients were in a varus direction. A procedural bias against patients with severe varus deformity may also have been involved, in which extra varus tibial cuts were performed on these patients to avoid the need for further additional cuts.

This study has several other limitations. Since the sample size of the study was relatively small, the patient group might not have completely represented the variations among patients undergoing TKA. Regarding radiographic evaluation, the use of closed-leg standing X-rays did not allow the evaluation of the dynamic joint line inclination during actual gait movement. In addition, there was no control group to compare the difference in the postoperative joint line between the KA and MA methods as previously reported [14]. Regarding the rotational alignment of the tibial component, there is no description of tibial rotational alignment in the rKA protocol reported by Vendittoli et al. [6–8] and it has not been established what index is appropriate for rKA. Therefore, Akagi's line was used in this patient group as the reference for rotational alignment of the tibial component [25]. Finally, while there have been no failures at this time, such as evident loosening or revision in the patient group, the long-term survivorship and relationship between joint line inclination and clinical outcomes remain undetermined. In fact, in this study 34 patients (46%) showed postoperative HKA deviations (more than ± 3˚) and 10 patients (14%) had postoperative MPTA exceeding the rKA target zone (within ± 5º), all of which were in a varus direction. However, Parratte and Bonner suggested that there is no impact on the long-term survival of implants when the TKA alignment deviates from the neutral position by more than 3º [29, 30]. Additionally, a recent study on KA TKA reported that postoperative alignment category of the tibial component and limb did not affect the 10-year implant survival [5]. Thus, catastrophic implant failure might be of lesser concern, but careful follow-up of these patients with alignment outliers is needed.

## Conclusions

The joint line during closed-leg standing after rKA TKA with CT-based PSI was within ± 3º of floor in 69% of the patients, with 86% of postoperative HKA within ± 3° of the planned alignment. The current study, despite a static verification, clarified how the joint line after rKA TKA was reproduced in the closed-leg long leg radiographs to mimic the limb position during gait. However, this imaging method is not well-established, and the lack of long-term survivorship and the relationship between joint line inclination and clinical outcomes represented limitations of this study.

## Data Availability

As no datasets were generated or analyzed during the current study, data sharing is not applicable to this article.

## Abbreviations

3D: Three-dimensional
CPAK: Coronal Plane Alignment of the Knee
CT: Computed tomography
HKA: Hip knee ankle angle
ICC: Inter-class correlation coefficient
JLOA: Joint line orientation angle
KA: Kinematic alignment
LDFA: Lateral distal femoral angle
MA: Mechanical alignment
MAE: Mean absolute error
MPTA: Medial proximal tibial angle
rKA: Restricted kinematic alignment
PSI: Patient-specific instrument
SD: Standard deviation
TKA: Total knee arthroplasty
